# The publication gender gap in US academic surgery

**DOI:** 10.1186/s12893-017-0211-4

**Published:** 2017-02-14

**Authors:** Claudia Mueller, Robert Wright, Sabine Girod

**Affiliations:** 1 Department of Surgery, Stanford University School of Medicine, 300 Pasteur Drive, Alway M116, Palo Alto, CA 94305 USA; 2 Department of Psychology, University of California, 900 University Avenue, Riverside, CA 92521 USA; 3 Department of Surgery, Stanford University School of Medicine, 1000 Welch Road #100, Palo Alto, CA 94304 USA

**Keywords:** Gender, Surgery, Hindex, Publication rate

## Abstract

**Background:**

Terms such as “glass ceiling” and “sticky floor” are still commonly used to describe women’s role in academic surgery. Despite continued efforts to address disparities between men and women in the field, gender inequalities persist.

**Methods:**

In this investigation we highlight gender differences in published surgical literature by both quantity and impact. Websites for departments of surgery of three academic centers were reviewed to assess the bibliometrics of publications by gender over a two-week period.

**Results:**

A one-way ANOVA showed a significantly higher H-index for men than women (*p* > .05). Further, one-way ANOVA showed significantly more articles published by men than women (*p* = .019). These differences are most dramatic at the rank of associate professor where the H-index for men is three times that of the women. The rank of full professor showed men had double the number of articles published.

**Conclusions:**

These findings align with the previous research that shows a disparity between males and females as they climb the academic ladder. Conducting and publishing research is a vital part of advancement in academic medicine. This study suggests that publication productivity may be a factor that hinders women from advancing within surgery compared to men. Continuing to explore and identify reasons for this gender difference in academic surgery may highlight ways to address the imbalance.

## Background

In spite of improvements in the hiring of women in Academic Medical Centers (AMCs) in recent years, there remain great inequities in the representation of women at the higher academic ranks [1, 2]. Indeed, terms akin to “glass ceiling” and “sticky floor” are still commonly used to describe women’s role in academic surgery [3].

Some of this may be attributed to gender-based hiring disparities of previous generations as well as to the documented high attrition of women at mid-career levels. However, these factors do not explain the persistence of gender differences in promotion rate at AMCs. Across a variety of specialties, both medical and surgical, women are promoted more slowly than their male colleagues [4, 5].

The delay in promotion could be related to several factors. Some studies have shown that women publish less, apply for fewer grants, and hold fewer leadership positions within their fields when compared to men [6–12]. These features might obviously lead to a slower rate of career advancement. However, it is also possible that some part of the delay might result less from a lack of productivity or effort and more from gaps in female faculty members’ understanding of the requirements and process of academic promotion.

In order first to establish whether women surgeons experience delays in their academic careers when compared to their male colleagues, we examined the publication records of female faculty members within the departments of surgery at three large academic centers. We hoped to highlight the differences in published literature by both amount and impact. Thus, we compared academic records on a number of factors including number of publications, academic rank, and time to promotion, as measured by years from fellowship. In order to assess citation impact, we calculated the *h*-index [13] for each faculty member and used this as a variable for gender comparison. We predicted that female faculty would have fewer publications and lower *h*-indices than their male counterparts while in lower ranks. However, we proposed that this effect might not be as apparent in the more advanced ranks, since we suspect that women may face higher standards, some of which may be self-imposed, before considering promotion.

## Methods

After an IRB waiver had been obtained, the websites for departments of surgery at three large academic centers (one on west coast and two on east) were reviewed. Surgeon gender, rank, and years from fellowship were determined from their online biographical information. Only full-time faculty members were included in the study. 212 faculty members were identified.. 52 of the surgeons were women and 160 were men. 74 surgeons held a rank of assistant professor (23 female, 51 male), 55 were associate professors (10 female, 45 male), and 77 were full professors (18 female, 59 male). Six others had indeterminate ranks (1 female, 5 male).

Over a two week span, data on number of articles published by each surgeon as well as the surgeon’s *h*-index were determined using the citation indexing service Web of Science™ (WoS).

## Results

All analyses were performed using univariate general linear model (ANOVA) or chi-square tests via IBM SPSS Statistics™.

Significant gender differences were identified on both measures of academic productivity that were assessed: number of publications and *h*-index. Male surgeons were found to publish significantly more articles than female surgeons (F 5.6, *p* < .05). The mean number of articles published by women was 33.7 compared with a mean of 52.3 articles by men (Table 1). Men also were noted to have a significantly higher *h*-index than their female counterparts (F 4.3, *p* < .05). Mean *h*-index for women was 12.6, while mean for men was 16.4 (Table 2). These findings remained significant across all ranks, from assistant to full professorship.

**Table 1:** Number of publications

Rank	Gender	Mean number of articles	Std. Deviation	*N*
Assistant	female	16.565	14.2057	23
	male	23.471	22.5808	51
	Total	21.324	20.5040	74
Associate	female	25.500	11.8533	10
	male	45.689	32.0687	45
	Total	42.018	30.3830	55
Professor	female	61.833	44.8491	18
	male	85.797	66.6007	59
	Total	80.195	62.7633	77
Other	female	2.000	.	1
	male	9.400	6.6182	5
	Total	8.167	6.6458	6
Total	female	33.673	35.0281	52
	male	52.262	53.0778	160
	Total	47.703	49.8377	212

**Table 2:** h-index

Rank	Gender	Mean	Std. Deviation	*N*
Assistant	female	6.391	4.3457	23
	male	8.627	5.7444	51
	Total	7.932	5.4202	74
Associate	female	11.300	3.7133	10
	male	14.933	7.1236	45
	Total	14.273	6.7562	55
Professor	female	21.944	13.0405	18
	male	25.203	12.6774	59
	Total	24.442	12.7522	77
Other	female	2.000	.	1
	male	5.000	2.2361	5
	Total	4.500	2.3452	6
Total	female	12.635	10.8628	52
	male	16.400	11.6620	160
	Total	15.476	11.5604	212

No significant gender differences were noted in mean years from fellowship at any rank (Table 3). Furthermore, no significant differences were noted between academic centers in terms of male to female faculty ratios at each academic rank, number of publications, h-indices, or number of post-fellowship years spent at each rank.

**Table 3:** Years from fellowship

Rank	Gender	Mean years	Std. Deviation	*N*
Assistant	female	5.048	3.3388	21
	male	5.833	4.1939	48
	Total	5.594	3.9457	69
Associate	female	12.400	6.6866	10
	male	12.946	6.7410	37
	Total	12.830	6.6605	47
Professor	female	22.471	8.4270	17
	male	23.691	7.7217	55
	Total	23.403	7.8501	72
Other	female	3.000	.	1
	male	2.400	1.8166	5
	Total	2.500	1.6432	6
Total	female	12.551	9.9038	49
	male	14.303	10.1251	145
	Total	13.861	10.0731	194

## Discussion

Our findings align with previous research that shows a persistent disparity between males and females as they climb the academic ladder. Overall, gender differences exist in the number of women compared with men who are employed by academic medical centers and who have attained higher academic ranks within those institutions.

In our study, we were able to identify significant and disturbing gender differences in terms of publication productivity. Female surgeons were found to publish fewer scientific articles, which also have less impact, when compared to their male counterparts. Furthermore, this was found to be true at each academic rank.

However, we did not find differences in the number of post-fellowship years that men and women have been in practice according to academic rank. Thus, although we are not able to determine from our data whether there may be differences in the timing of promotion consideration, it does not appear that women are spending significantly more years within lower academic ranks than men.

One interpretation of this finding is that there exists some form of reverse discrimination at these academic centers, which allows women to be promoted with less scholarship than men. However, given overall stringent promotion standards and the high absolute number of publications by women in the upper ranks (25 for associate and 62 for full professors), this does not seem to be the most likely explanation. Instead, we propose that the careers of female surgeons are more likely to encompass other academic pursuits, such as teaching and hospital-service roles, which may contribute toward promotion. These activities, while vital to any academic medical center, are less easily quantified by the available internet resources of websites and citation indices. They are also perhaps less commonly pursued by male surgeons, who may be more likely to rely on the traditional notion of publication rate as the ultimate marker of advancement, thus resulting in the higher number of articles published by men in our sample.

Of course, our study has some significant limitations. First, our examination focuses on only three academic centers, which, although diverse in faculty, may limit the generalizability of our findings. In addition, we did not distinguish between order of authorship within our analyses. Including this detail might allow us to refine our conclusions about gender issues surgical within publishing practices. Finally, our use of departmental websites limits the type of information we have on each faculty member so that we are missing details such as number of years to promotion, percentage clinical effort, and amount of dedicated research time. The availability of this data would likely greatly enhance our ability to understand the gender differences we have identified.

## Conclusions

In sum, we were able to identify significant gender differences in rate and impact of publications, with women showing worse productivity than men in surgery. We propose that future studies on gender inequalities should include assessments on a larger number of medical centers and should include more detailed information on both academic output (e.g., order of authorship) and faculty career tracks. In addition, we hope to focus on closer comparisons of promotion factors between male and female academic surgeons. We strongly hope that such investigations will improve our understanding of gender inequalities within academic surgery with the ultimate aim of identifying mechanisms of support for advancement, which can be used by male and female faculty alike.

## References

[1] Thomas PA, Diener-West M, Canto MI, Martin DR, Post WS, Streiff MB (2004). Results of an academic promotion and career path survey of faculty at the Johns Hopkins University School of Medicine. Acad Med.

[2] Committee on maximizing the potential of women in academic science and engineering, National Academy of Sciences, National Academy of Engineering, and Institute of Medicine, (2006). Beyond bias and barriers; fulfilling the potential of women in academic science and engineering. Available: http://nap.edu/catalog/11741.html.

[3] Zhuge Y, Kaufman J, Simeone DM, Chen H, Velazquez OC (2011). Is there still a glass ceiling for women in academic surgery. Ann Surg.

[4] Tesch BJ, Wood HM, Helwig AL, Nattinger AB (1995). Promotion of women physicians in academic medicine. Glass ceiling or sticky floor?. JAMA.

[5] Wright AL, Schwindt LA, Bassford TL, Reyna VF, Shisslak CM (2003). St Germain, P. A., & Reed KL. Gender differences in academic advancement: patterns, causes, and potential solutions in one US College of Medicine. Acad Med.

[6] Wagner AK, McElligott J, Chan L, Wagner EP, Segal NA, Gerber H (2007). How gender impacts career development and leadership in rehabilitation medicine: a report from the AAPM&R research committee. Arch Phys Med Rehabil.

[7] Taira BR, Jahnes K, Singer AJ, McLarty AL (2008). Does reported funding differ by gender in the surgical literature. Ann Surg.

[8] Sidhu R, Rajashekhar P, Lavin VL, Parry J, Attwood J, Holdcroft A, Sanders DS (2009). The gender imbalance in academic medicine: a study of female authorship in the United Kingdom. J R Soc Med.

[9] Schroen AT, Brownstein MR, Sheldon GF (2004). Women in academic general surgery. Acad Med.

[10] Sonnad SS, Colletti LM (2002). Issues in the recruitment and success of women in academic surgery. Surgery.

[11] Waisbren SE, Bowles H, Hasan T, Zou KH, Emans SJ, Goldberg C, Christou H (2008). Gender differences in research grant applications and funding outcomes for medical school faculty. J Womens Health (Larchmt).

[12] Wolfinger NH, Mason MA, Goulden M (2008). Problems in the pipeline: gender, marriage, and fertility in the ivory tower. J High Educ.

[13] Hirsch J (2005). An index to quantify an individual's scientific research output. PNAS.

